# Aldehyde dehydrogenase inhibitors promote DNA damage in ovarian cancer and synergize with ATM/ATR inhibitors

**DOI:** 10.7150/thno.51885

**Published:** 2021-01-20

**Authors:** Edward Grimley, Alexander J. Cole, Thong T. Luong, Stacy C. McGonigal, Sarah Sinno, Dongli Yang, Kara A. Bernstein, Ronald J. Buckanovich

**Affiliations:** 1 Division of Gynecologic Oncology, Department of Obstetrics and Gynecology, UPMC Hillman Cancer Center and the Magee-Womens Research Institute, University of Pittsburgh, Pittsburgh, PA, USA.; 2 Dept of Microbiology and Molecular. Genetics, University of Pittsburgh School of Medicine, UPMC Hillman Cancer Center, Pittsburgh, PA, USA.; 3 Department of Internal Medicine, University of Pittsburgh, Pittsburgh, PA, USA.

**Keywords:** aldehyde dehydrogenase, DNA damage, Ovarian cancer, ATM, ATR

## Abstract

**Rationale**: Aldehyde dehydrogenase (ALDH) enzymes are often upregulated in cancer cells and associated with therapeutic resistance. ALDH enzymes protect cells by metabolizing toxic aldehydes which can induce DNA double stand breaks (DSB). We recently identified a novel ALDH1A family inhibitor (ALDHi), 673A. We hypothesized that 673A, via inhibition of ALDH1A family members, could induce intracellular accumulation of genotoxic aldehydes to cause DSB and that ALDHi could synergize with inhibitors of the ATM and ATR, proteins which direct DSB repair.

**Methods**: We used immunofluorescence to directly assess levels of the aldehyde 4-hydroxynonenal and comet assays to evaluate DSB. Western blot was used to evaluate activation of the DNA damage response pathways. Cell counts were performed in the presence of 673A and additional aldehydes or aldehyde scavengers. ALDH inhibition results were confirmed using ALDH1A3 CRISPR knockout. Synergy between 673A and ATM or ATR inhibitors was evaluated using the Chou-Talalay method and confirmed *in vivo* using cell line xenograft tumor studies.

**Results**: The ALDHi 673A cellular accumulation of toxic aldehydes which induce DNA double strand breaks. This is exacerbated by addition of exogenous aldehydes such as vitamin-A (retinaldehyde) and ameliorated by aldehyde scavengers such as metformin and hydralazine. Importantly, ALDH1A3 knockout cells demonstrated increased sensitivity to ATM/ATR inhibitors. And, ALDHi synergized with inhibitors of ATM and ATR, master regulators of the DSB DNA damage response, both *in vitro* and *in vivo.* This synergy was evident in homologous recombination (HR) proficient cell lines.

**Conclusions**: ALDHi can be used to induce DNA DSB in cancer cells and synergize with inhibitors the ATM/ATR pathway. Our data suggest a novel therapeutic approach to target HR proficient ovarian cancer cells.

## Introduction

Ovarian cancer is a highly lethal disease. As DNA damage repair deficiency is a hallmark of many ovarian high grade serous ovarian cancers 1, 2, DNA damaging agents, such as platinum chemotherapies, have been a core of ovarian cancer therapy 3. Importantly, recent studies with PARP inhibitors, which can increase DNA damage in BRCA mutated cancers, can significantly improve the survival of patients with BRCA mutated tumors 4. Despite this important success, PARP inhibitors have much more limited activity for most patients (non-BRCA carriers) with ovarian cancer 5. Thus new, additional, therapeutic targets are needed, particularly for the patients with homologous recombination (HR) proficient tumors.

All living organisms are required to respond to oxidative stresses caused by normal cellular processes, such as, mitochondrial respiration, xenobiotic metabolism and inflammation. These oxidative stresses promote the formation of reactive oxygen species (ROS), which cause oxidative degradation of lipid membranes resulting in the generation of >200 types of aldehydes. Due to their electrophilic nature, aldehydes are highly toxic and reactive, capable of forming adducts with DNA, RNA and proteins, causing enzyme inactivation, impaired homeostasis, DNA damage and apoptosis 6. Aldehyde mediated DNA damage therefore acts a potent activator of a wide range of DNA damage repair pathways, including Fanconi anemia (FA) pathway, Trans-lesion DNA synthesis, base excision repair (BER), nucleotide excision repair (NER), fork protection complex, homologous recombination (HR) and ATR-dependent cell cycle checkpoint activation 7.

The Aldehyde dehydrogenase (ALDH) superfamily, consisting of 19 enzymes in humans, possess NAD(P)+-dependent enzymatic activity responsible for catalyzing the oxidation of aldehydes to carboxylic acids 8, and thereby detoxify and protect cells from aldehydes and ROS accumulation 9, 10. These enzymes have diverse function in a wide range of biological processes, including vitamin-A metabolism into retinoic acid. Recently, ALDH enzymes have also been shown to play vital role in cancer stem cells (CSCs) in a variety of cancer types, including ovarian cancer 11, and hence represent a potential new therapeutic target 12. The most common ALDH family associated with cancer stem cells are the ALDH1A family, which comprise; ALDH1A1, ALDH1A2 and ALDH1A3 13. Consequently, considerable efforts have been made to target these enzymes for anticancer therapy.

Numerous studies have also reported efficacy of various ALDH inhibitors (ALDHi) in a variety of different cancer types 14-19. Recently, we reported on the development and efficacy of the pan-ALDH1A family inhibitor 673A to induce death in ovarian CSC 20. We showed that death was in part due to ALDH inhibition mediated changes in downstream retinoic acid (RA) mediated transcription. In this study, we evaluate the upstream effects of ALDH inhibition on the accumulation of toxic aldehydes. We demonstrate inhibition of ALDH in ovarian cancer cell lines using 673A results in a significant build-up of aldehydes within the cell. Aldehyde build-up results in DNA damage, and a significant decrease in cell viability. Similarly, ALDH1A3 knockout in an ALDH1A3 dominant cell line reduced cancer cell viability and significantly restricted cancer initiation and growth. Importantly, both ALDH knockout and 673A treatment of ovarian cancer cell lines synergized with DNA damage checkpoint inhibitors, AZD1390 (ATM inhibitor) and AZD6738 (ATR inhibitor), resulting in a synergistic decrease in cell viability *in vitro* and tumor growth *in vivo*. Combined, this work identifies a novel combination therapy for cancer.

## Methods

### Cell culture

The cell line was obtained from Susan Murphy at Duke University (Durham, North Carolina, USA). The OVCAR5 (possessing a KRAS mutation) and HEY1 cell lines were purchased from ATCC. OVCAR4 cells were a gift from Deborah Marsh from the University of Sydney (Sydney, New South Wales, Australia). All cells were cultured in RPMI-1640 medium with 10% FBS and 1% penicillin/streptomycin at 37 ° C and 5% CO_2_.

### Cell viability and synergy assays

Cells were grown in RPMI 1640 media (Corning) containing 2 mM glutamine, 10% FBS (Sigma), and 1× Pen/Strep (Gibco). When the cells reached 80% confluency, they were harvested by trypsinization, washed with PBS, and reseeded in 96-well plates at 2000 cells/well. The cells were allowed to recover for 24 h, vehicle, inhibitor(s) and/or aldehydes were added, and the cells were put back into the incubator for 96 h. Following the 96-hr incubation, the media was removed and a 1× solution of Cell-Titer Glo 2.0 (Promega) was added. The plates were mixed and allowed to incubate at room temperature for 30 min before luminescence was read on an Infinite M Plex (Tecan) plate reader. All assays were performed at least 3 times with three technical replicates. Normalized viability was calculated by comparing the luminescence of drug-treated wells to vehicle-treated wells and expressed as a percentage. The percentage of viable cells was graphed in Prism 7 and all data are displayed as mean ± SD. Synergy was assessed using Chou-Talalay method and the CompuSyn program 21.

### Neutral Comet assay

200,000 cells were seeded in a 6-well plate for each condition and grown overnight at 37 º C. After 18 hrs, fresh medium containing DMSO or ALDH1Ai (673A; at 0.5 μM or 1 μM) was added for 24 hrs before harvesting. All subsequent steps were performed in low-light condition. Cells were trypsinized and washed before being resuspended in PBS at 100,000 cells per mL. Cells were resuspended 1:10 in molten LMAgarose (Trevigen) and 30 µl was pipetted onto a CometSlideTM (Trevigen). The slide was chilled to 4 º C and all subsequent buffers and electrophoresis unit (Trevigen Comet Assay ES II) were also chilled. The slide was immersed in CometAssay lysis solution (Trevigen) for 1 hr at 4 º C and subsequently incubated in neutral electrophoresis buffer (100 mM Tris base, 300 mM sodium acetate) at 4 º C for 30 min. Electrophoresis was performed at 21 volts for 45 min at 4 º C. The slide was transferred to DNA precipitation buffer (6.7 mL of 7.5 M ammonium acetate and 43.3 mL 95% ethanol) for 30 min at room temperature (RT), then immersed in 70% ethanol for 30 min at RT, and dried overnight at RT. Subsequently, the slide was stained using SYBR Gold solution (1 μL of SYBR in 30 mL Tris-EDTA buffer, pH 7.5) for 30 min at RT and dried overnight at RT. Comet tails were analyzed using an epifluorescence Nikon TiE inverted microscope and tail moments were analyzed using CometAssay IV software (Instem). The experiment was performed with a biological replicate and in duplicate.

### ALDEFLUOR assay

Cells were grown in RPMI 1640 media (Corning) containing 2 mM glutamine, 10% FBS (Sigma), and 1× Pen/Strep (Gibco). When the cells reached 80% confluency, they were harvested by trypsinization and assayed for ALDH activity using the Aldefluor assay (STEMCELL Technologies) as previously described 22. Briefly, the cells were washed with PBS, resuspended in Aldefluor buffer and Aldefluor reagent was added. The cells were quickly mixed and evenly distributed into 1.5 mL Eppendorf tubes containing inhibitor or vehicle and incubated for 30 min at 37 ° C. Cells were washed, resuspended in fresh Aldefluor buffer that had been kept on ice until they were analyzed on a CytoFLEX S flow cytometer (Beckman Coulter). The percent of control values were calculated using the percentage of Aldefluor positive cells for a particular sample and the percentage of Aldefluor positive cells in the control sample (vehicle-treated). The percentage of Aldefluor positive cells was graphed in Prism 7 (GraphPad) and is displayed as mean ± SD. The Two-way ANOVA with Tukeys multiple comparison test within Prism 7 (GraphPad) was used to determine statistical significance between samples treated with compound or vehicle.

### Western blotting

For Western blot analysis, the media were removed from test plates, and cells were lysed in RIPA buffer (Pierce). Lysates were briefly sonicated, centrifuged, and the cleared lysate was transferred to a clean Eppendorf tube. The protein concentration was determined by BCA assay (Pierce). Equal amounts of protein were separated on 4-12% Bis-Tris gels (Life Technologies) and transferred to PVDF membranes. Membranes were blocked with 5% non-fat milk in TBS for one hour before primary antibodies were added. Primary antibodies (FL-PARP, CL-PARP, KU-70, KU-80, pChk1, pChk2, γ -H2AX and b-Actin) were added in 1% non-fat milk in TBST and incubated at 4 ⁰ C overnight with gentle rocking. Membranes were washed 3× for 10 min with TBST before secondary antibodies were added. immunoblotted with the indicated antibody. Secondary antibodies were added in 1% non-fat milk in TBST and incubated at room temperature for one hour with gentle rocking. Membranes were washed 3× for 10 min with TBST and 2× for 5 min with TBS before chemiluminescent development.

### Isolation of RNA and reverse transcriptase-quantitative PCR (RT-qPCR)

Cells were lysed using RNeasy Mini kit from Qiagen (Hilden, Germany) according to the manufacturer's instruction. After removal of contaminating DNA using DNA-free (Invitrogen), extracted RNA was quantified and quality was assessed by 260/280 absorbance ratio using a NanoDrop-1000 spectrophotometer (Fisher-Thermo). All 260/280 ratios were above 1.9. 1 μg of RNA served as the template for reverse transcription using the High-capacity cDNA reverse transcription kit (Applied Biosystems, Warrington, UK) according to the manufacturer's instructions. The RT product was used for PCR using 250 nM concentrations for forward and reverse gene-specific primers (**Table S1**).

Reactions were run in duplicate using 384 well plates with 25 ng of cDNA per 10 μL of reaction mixture using SYBR Green PCR Master Mix (Applied Biosystems, Warrington, UK) and analyzed using a BioRad CFX 384 Real time system C1000 touch thermal cycler (BioRad Hercules, CA). Dissociation curves were run on all reactions, and samples were normalized to YWHAZ. The ΔΔCt method was used to determine relative gene expression.

### Immunofluorescence (IF)

IF was performed on paraffin embedded sections (5 µm) as previously described 23. Briefly, sections were deparaffinized in xylene and ethanol before antigen retrieval in sodium citrate buffer (10 mM, pH 7.5) for 20 min at 90 ° C. Cultured cells were plated on coverslips, cultured overnight and treated the next day. At collection cells were fixed in 4% paraformaldehyde and permeabilized in 0.5% Triton X-100/PBS. Acrolein antibody (Invitrogen MA527553 Waltham, MA) was used at 1:50 and anti-4-Hydroxynonenal antibody (ab46545 from Abcam Cambridge, MA) was used at 1:100 and incubated overnight at 4 ° C, anti-γH2A.X (Cell Signalling Rabbit mAb#9718) was used at 1:500. Secondary goat anti-rabbit Daylight 488 (Fisher PI35552) was used at 1:500 and incubated for 45 min in the dark at room temperature. Following 3 washes, slides were then mounted with DAPI mounting media. Images were captured using the Leica DM4 B upright microscope (Leica Chicago, IL).

### ALDH1A3 CRISPR knockout

OVCAR5 cells were transduced with one of three human sgRNA CRISPR All-in-One Lentivirus (Applied Biological Materials Inc) targeting ALDH1A3 at MOI of 5. The cells were subsequently subjected to 7 days of puromycin selection before being clonally selected using limiting dilution. Knockout was confirmed using qPCR and ALDEFLUOR assays.

### *In vivo* therapeutic studies

OVCAR5 cells were grown in RPMI 1640 media (Corning) containing 2 mM glutamine, 10% FBS (Sigma), and 1× Pen/Strep (Gibco). When the cells reached 80% confluency, they were harvested by trypsinization, washed and resuspended in cold PBS 1:1 with Matrigel (Corning) as previously described were mixed and 100 µL of this mixture was loaded into chilled insulin syringes such that each syringe carried 50,000 cells 24. This cell suspension was injected subcutaneously into the axillary region of nude mice. The cells were given 3 days to engraft before treatments were started. Mice were injected daily with vehicle or 20 mg/kg 673A and/or given vehicle, 20 mg/kg AZD1390, or 50 mg/kg AZD 6738 by oral gavage daily. Tumors were measure by caliper and volume was calculated (L*W*W/2). Tumor growth curves were graphed in Prism 7 (GraphPad) and is displayed as mean ± SD.

## Results

### The ALDH1A family inhibition causes a buildup in toxic aldehydes which induce DNA damage and cytotoxicity

**Figure 1:**
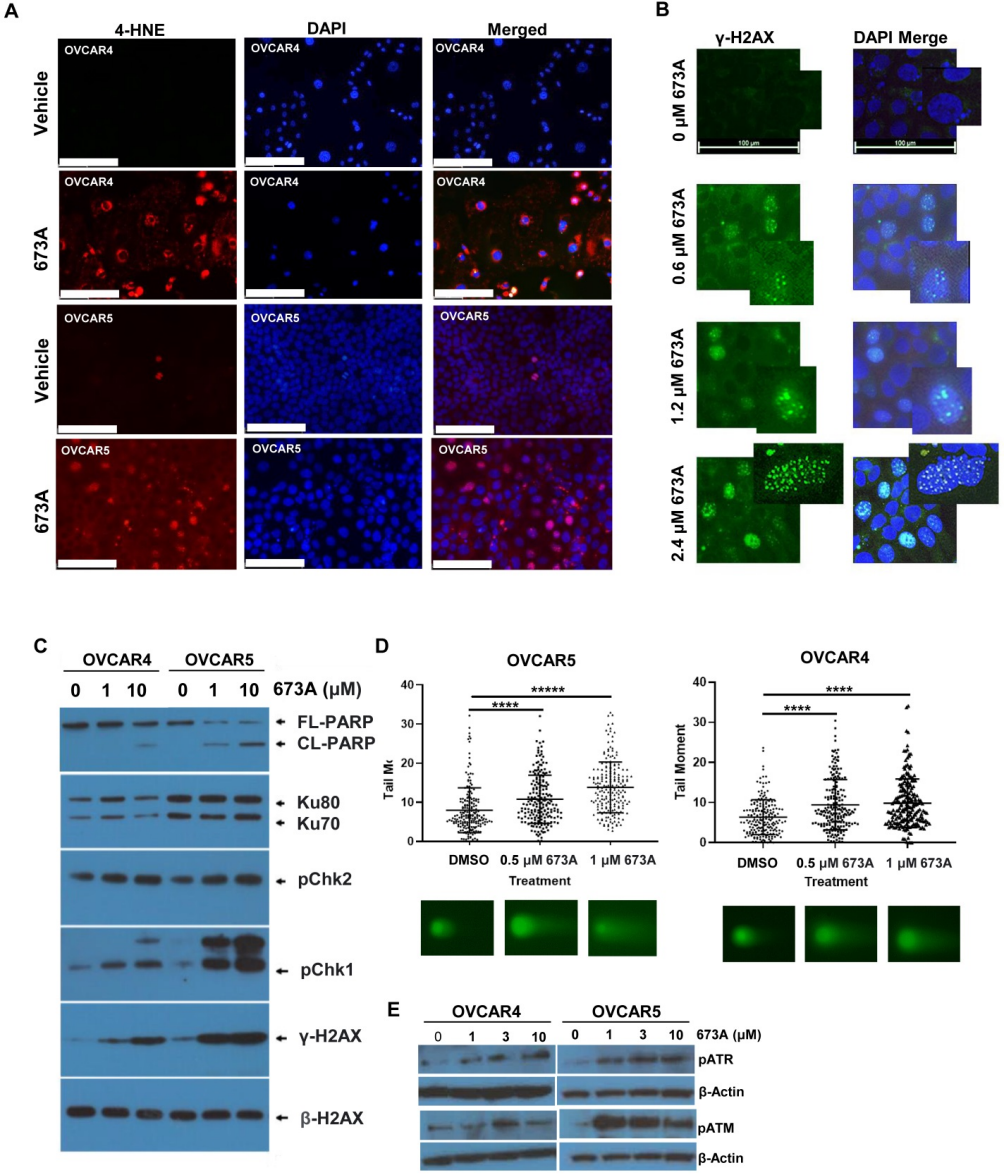
** The aldehyde dehydrogenase inhibitor 673A causes a buildup in aldehydes, a decrease in cell viability and an increase in DNA damage. A)** IF for 4-HNE in OVCAR5 and OVCAR4 cells treated with or without 673A. **B)** IF of g-H2AX foci in cells treated with 0, 0.6, 1.2 and 2.4 µM 673A. A high-power inset is included to show foci in greater detail. **C)** Western Blot of yH2AX, pChk1, pChk2, and cleaved-PARP, in OVCAR4, OVCAR5 cells treated with 0, 1 µM or 10 µM 673A. **D)** Western blot for Neutral comet assays were performed in OVCAR4 and OVCAR5 cell lines treated with DMSO or the indicated concentrations of 673A. 200 tail moments were counted and graphed from two independent experiments with mean and standard deviation plotted. Significance was determined by t-test. **** indicates p-value of <0.0001. **E)** Western blot pATM and pATR in OVCAR4 and OVCAR5 cells treated with 673A (0, 1, 3 and 10 µM). Scales bars are 100mm.

Aldehyde dehydrogenase enzymes function to metabolize aldehydes to form carboxylic acids allowing for their elimination, thereby facilitating cellular detoxification. To evaluate the effects of inhibiting the ALDH1A family of enzymes on aldehyde levels, we inhibited aldehyde dehydrogenase using the pan-ALDH1A inhibitor 673A and evaluated 4-hydroxynonenal (4-HNE) levels via IF. For our studies, we used OVCAR4 cells (p53 mutant, CCNE high, homologous recombination proficient cells) 25, and OVCAR5 cells a KRAS mutant homologous recombination proficient cell line. These cell lines were chosen to represent a spectrum of response to 673A, with OVCAR5 most sensitive and OVCAR4 moderately responsive (**Figure S1A**). Compared with vehicle-treated cells, treatment of the cancer cell lines OVCAR4 and OVCAR5 with 673A (1 and 2.5 µM respectively) resulted in a dose dependent increase in 4-HNE levels in both cell lines (**Figure 1A**). 4-HNE increase was dose dependent (**Figure S1B**).

Aldehydes can cause DNA damage through the formation of DNA adducts 26. To determine if this build up in aldehydes following 673A treatment resulted DNA damage, we evaluated γ-H2AX foci as a marker of DNA damage 673A. 673A treatment resulted in a dose dependent increase in γ-H2AX foci (**Figure 1B**). To further interrogate this response, western blotting for the DNA damage markers γ-H2AX, pChk1, and pChk2 was conducted on cancer cell lines (OVCAR4 and OVCAR5) treated with 0 µM, 1 µM or 10 µM 673A for 12 h (**Figure 1C**). β-Actin was used as a loading control. γ-H2AX, pChk1 and pChk2 increased in a dose dependent manner following 673A treatment, together confirming an increase in DNA damage following 673A treatment. A concomitant increase in the ratio of cleaved-PARP to total-PARP levels indicated the increase in DNA damage is associated with increased cell death. HEY1 cells (p53 mutant, KRAS mutant), were tested in parallel to assess if response was related to the KRAS mutation. Response to 673A did not appear dependent on the KRAS mutation, as the KRAS mutant HEY1 cells were most resistant to 673A induced DNA damage (**Figure S1C**).

We next evaluated 673A treatment on DNA damage, using a neutral comet assay specific for DNA double strand breaks**.** DNA tail moment, indicative of DNA double-strand breaks, significantly increased in a dose dependent manner following 673A treatment compared to vehicle (P<0.0001)** (Figure 1D, Figure S2)**. Consistent with DNA double strand breaks, western blot analysis showed an increase in phosphorylation of the ataxia telangiectasia and Rad3-related kinase (ATR), and phosphorylated Ataxia telangiectasia mutated kinase (ATM) DNA damage repair/checkpoint proteins (**Figure 1E**).

### ALDH1A family inhibition induced death can be exacerbated by exogenous aldehydes and ameliorated by aldehyde scavengers

**Figure 2:**
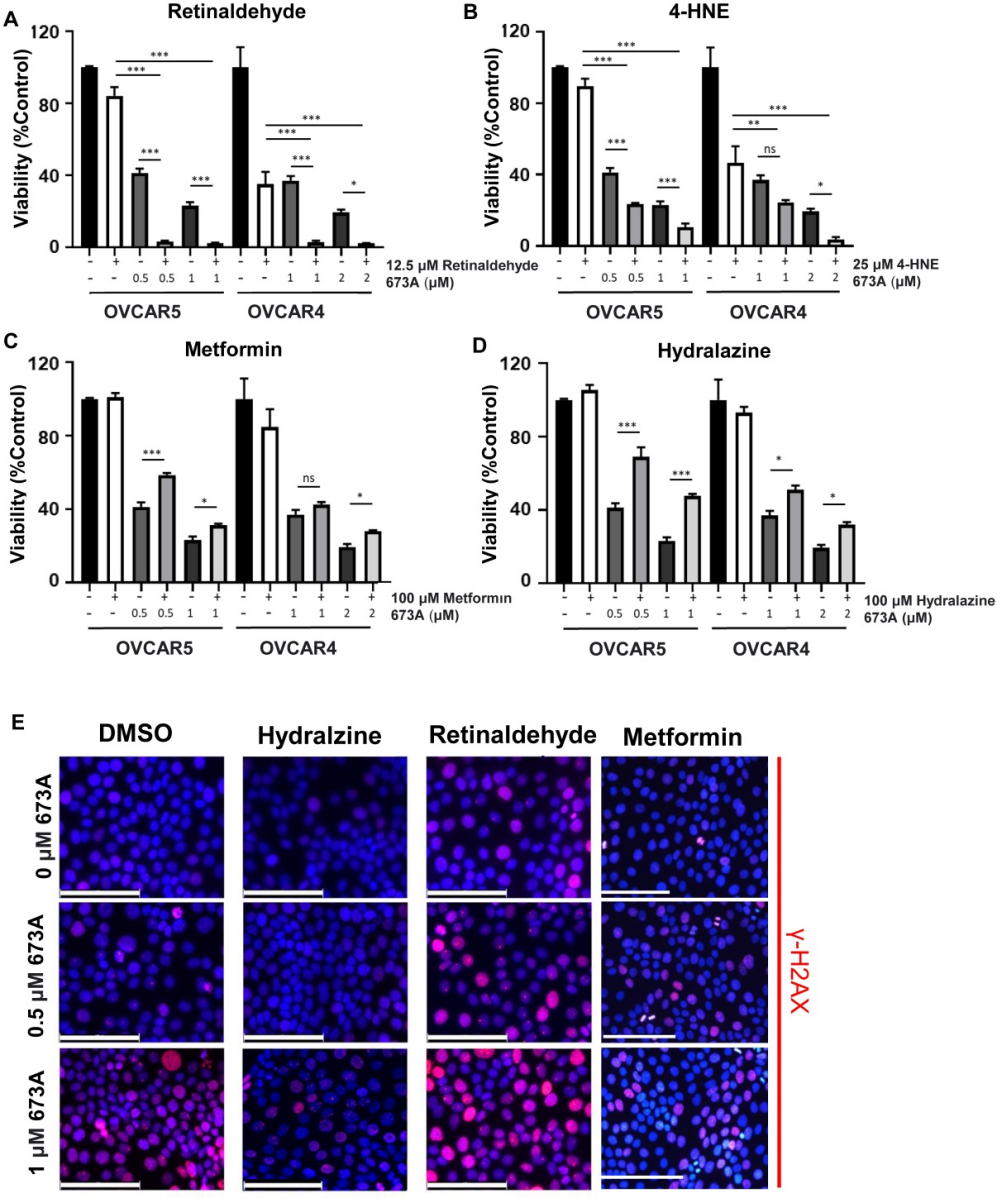
** Co-treatment of 673A and aldehydes reduce cellular viability.** Cell viability of OVCAR5 and OVCAR4 cells treated with 673A alone or in combination with **A)** Retinaldehyde **B)** 4-HNE, **C)** Metformin or **D)** Hydralazine. All assays were repeated at least three times and included three technical replicates. Date represent means with errors bars indicating standard deviation. * p ≤ 0.05, ** p ≤ 0.01, *** p ≤ 0.001. **E)** IF Images of g-H2AX foci in cells treated with 673A, hydralazine, retinaldehyde or the combination. Scales bars are 100mm.

To further confirm a role for aldehydes we tested the impact of the addition of exogenous aldehydes or aldehyde scavengers on 673A mediated death. To further investigate the relationship between DNA damage and the increase in levels of toxic aldehydes, ovarian cancer cells were treated with 673A alone, 673A in combination with retinaldehyde or 4-HNE, or 673A combined with the aldehyde scavengers metformin or hydralazine. The addition of either retinaldehyde or 4-HNE demonstrated to 673A significantly increased cell death (**Figure 2A-B**). In contrast, both the aldehyde scavengers metformin and hydralazine reduced 673A driven cytotoxicity (**Figure 2C-D).** Calculation of combination indices (<1 being synergistic, >1 being antagonistic) confirmed synergy of aldehydes and 673A, and antagonism of metformin scavengers and 673a (**Table 1**). Similarly, IF imaging demonstrated that addition of aldehydes exacerbated γ-H2AX foci formation in cells, while addition of scavengers reduced γ-H2AX foci formation in cells (**Figure 2E**). Western blot for γ-H2AX and cleaved PARP showed similar results (**Fig S3A**).

To confirm the effect of 673A on γ-H2AX was a result of specific inhibition of the ALDH1A family, we tested the two additional pan ALDH1A inhibitors, UM122 and UM548 14. Both similarly resulted in a significant increase in γ-H2AX levels (**Figure S3B**). Combined, this data suggests ALDHi contribute to cancer cell death via an accumulation of DNA damaging aldehydes which induce double strand DNA breaks. This data supports concept of DSB induction as a result of aldehyde accumulation, causing a reduction in cell viability.

### ALDH1A3 knockout increases cancer cell sensitivity to aldehydes

Based on The Cancer Genome Atlas (TCGA) data set 1, gene alterations in ALDH1A family member occurs in 20.57% of high grade serous ovarian cancers (HGSOC). Suggesting ALDH1A enzymes perform critical functions to promote tumorigenesis, deletions are rare and gene amplification account for the majority of these mutations (**Figure S4**). Due to the ability of aldehydes to promote DNA damage and cell death, amplification of ALDH family member may protect cancer cells from the DNA damaging effects of aldehyde accumulation. To investigate this and confirm on-target activity of 673A, we deleted ALDH1A3 (the dominant ALDH1A family member -**Table S2**) in OVCAR5 cells using CRISPR**.** The OVCAR5 cells line was selected for this experiment due to its high proportion of ALDH positive cells 20.

**Figure 3:**
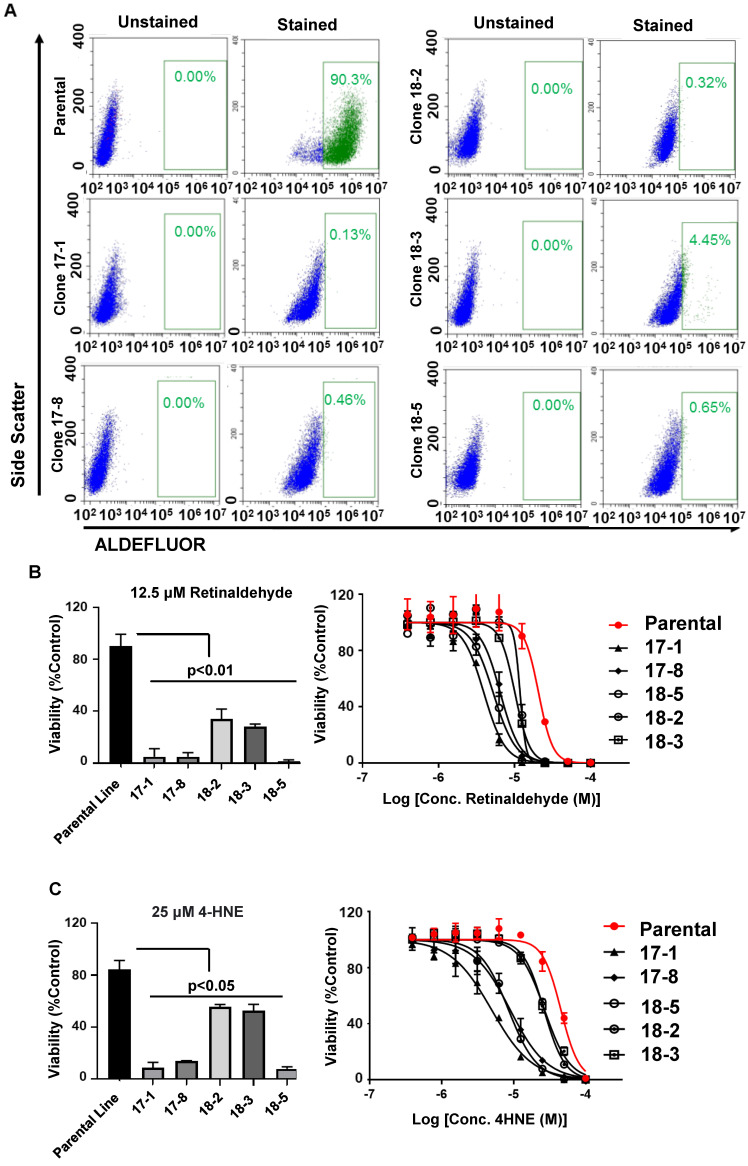
** Functional validation of ALDH1A3-deleted OVCAR5 cells. A)** Parental and ALDH1A3 CRISPR clones stained with an ALDEFLUOR assay and gated for ALDH positive cells. **B)** Bar graph of the viability of parental OVCAR 5 and OVCAR5 ALDH1A3 knockout clones treated with 12.5 µM retinaldehyde, and viability curves of ALDH1A3 CRISPR clones treated with increasing concentrations of retinaldehyde. **C)** Bar graph of the viability of parental OVCAR 5 and OVCAR5 ALDH1A3 knockout clones treated with 25 µM 4-HNE, and viability curves of ALDH1A3 CRISPR clones treated with increasing concentrations of 4-HNE. All assays were performed at least three times.

Multiple individual OVCAR5 ALDH1A3 knockout clones resulted were isolated and characterized. qRT-PCR demonstrated a 3.79-572-fold reduction in ALDH1A3 mRNA expression (**Table S3**). Functional loss of ALDH1A3 protein activity was validated by performing an endogenous ALDEFLUOR activity assay (**Figure 3A**). Normal parental OVCAR5 cells were >90% ALDH bright. While OVCAR5 CRISPR clones cell clones ranged from 4.45%-0.13% ALDH bright cells, closely reflecting their mRNA expression levels of ALDH1A3.

To test the impact of ALDH1A3 loss on aldehyde induced toxicity, the OVCAR5 parental cells and ALDH1A3 CRISPR clones were treated with either a fixed low dose of Retinaldehyde or increasing concentrations of Retinaldehyde. All OVCAR5 ALDH1A3 CRISPR clones were significantly more sensitive to retinaldehyde demonstrating between a ~2.5-10-fold increase in cell death (**Figure 3B**). The clone, 18-3, which retained some residual ALDH1A3 expression and ALDEFLUOR activity was mildly less sensitive to aldehyde induced death. Similarly, treatment of the parental cell line and OVCAR5 ALDH1A3 CRISPR clones with 25 µM 4-HNE or increasing dose of 4-HNE resulted in significantly more death in the knockout cells (**Figure 3C**). In summary, this data suggests expression of ALDH1A3 is essential for these cells to overcome aldehyde induced DNA damage and cell death.

### Cells with decreased ALDH activity are more sensitive to inhibitors of the DNA damage repair checkpoint pathways

**Figure 4:**
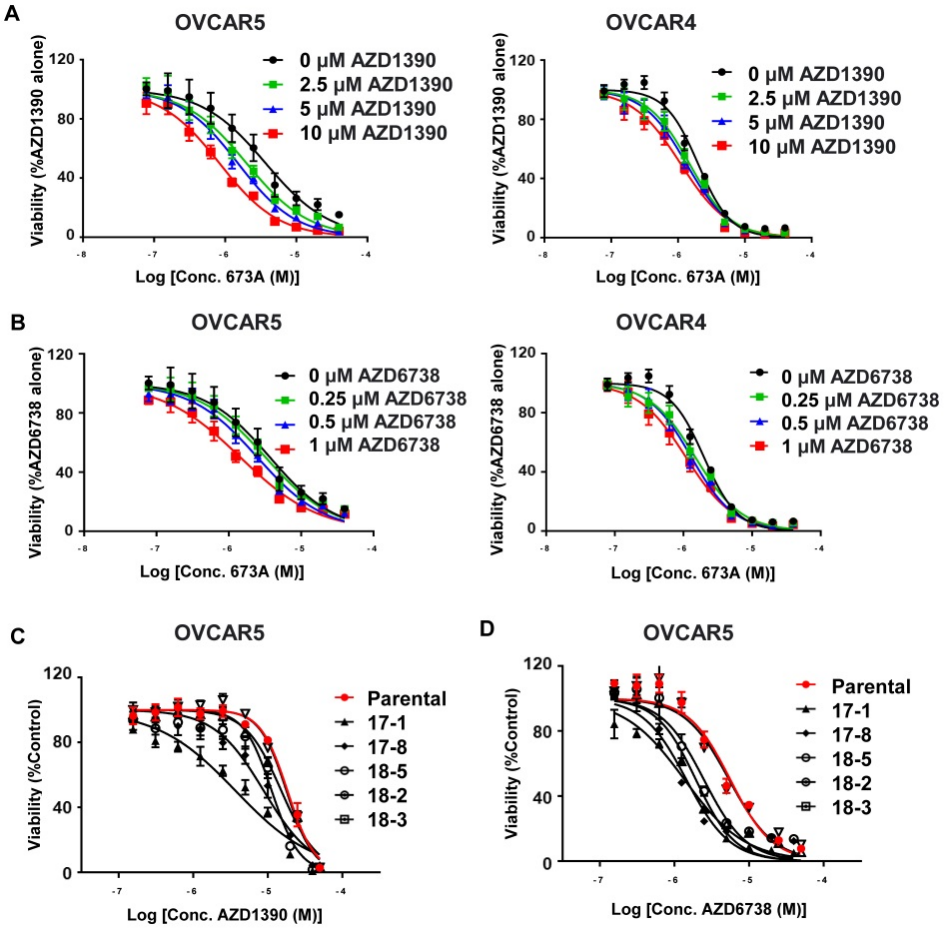
** Cancer cells lacking ALDH activity have increased sensitivity to ATM (AZD1390) and ATR inhibitors (AZD6738). A)** Viability curves for OVCAR5 and OVCAR4 cells treated with 2.5 µM, 5 µM, and 10 µM ATM (AZD1390) inhibitors and/or 673A. **B)** Viability curves for OVCAR5 and OVCAR4 cells treated with 0.25 µM, 0.5 µM, and 1 µM ATR (AZD6738) inhibitor and/or 673A. **C) and D)** Viability curves for OVCAR5 cells and several OVCAR5 ALDH1A3 CRISPR KO clones treated with ATM (AZD1390) or ATR (AZD6738) inhibitors and/or 673A. All assays were repeated at least three times, with data points representing averages and error bar standard deviations.

The Fanconi anemia (FA) and homologous recombination (HR) pathways are essential for repair of aldehyde-induced genotoxicity 27. We hypothesised that inhibiting the ALDH1A family using 673A treatment (or ALDH1A3 knockout) would increase aldehyde induce DNA damage which could result in an increase in the efficacy of drugs that inhibit DNA damage repair. In particular, as western blot indicates activation of the DNA damage response pathway orchestrated by ATM and ATR, 28 we focused on inhibitors of ATM and ATR. To test this, we treated OVCAR5 and OVCAR4 cells with the 673A and ATM (AZD1390) or ATR (AZD6738) inhibitors and measured cell viability. Treatment of OVCAR5 and OVCAR4 cells with 673A in combination with varying doses of AZD1390 (2.5 µM, 5 µM, and 10 µM) resulted in a significant reduction in cell viability compared with 673A alone (**Figure 4A**). Similarly, treatment of OVCAR5 and OVCAR4 cells with 673A in combination with varying doses of AZD6738 (0.25 µM, 0.5 µM, and 1 µM) also resulted in a significant reduction in cell viability compared with 673A alone (**Figure 4B**). Calculated combination indices confirmed synergy of 673A with both compounds (**Table 2**).

To confirm the importance of ALDH on the ability of cells to cope with DNA damage, we treated the OVCAR5 ALDH1A3 CRISPR deletion clones with ATM (AZD1390) or ATR (AZD6738) inhibitors. All OVCAR5 ALDH1A3 CRISPR deletion clones treated with either ATM or ATR inhibitors demonstrated a significant decrease in cell viability compared to the parental cell line (**Figure 4C, D**). Together, this data demonstrates expression of ALDH is important for a cell's ability to overcome aldehyde induced DNA damage and prevent cell death.

### Combined treatment with ALDHi and ATM or ATR inhibitors results in reduced tumor burden in a mouse xenograft model

**Figure 5:**
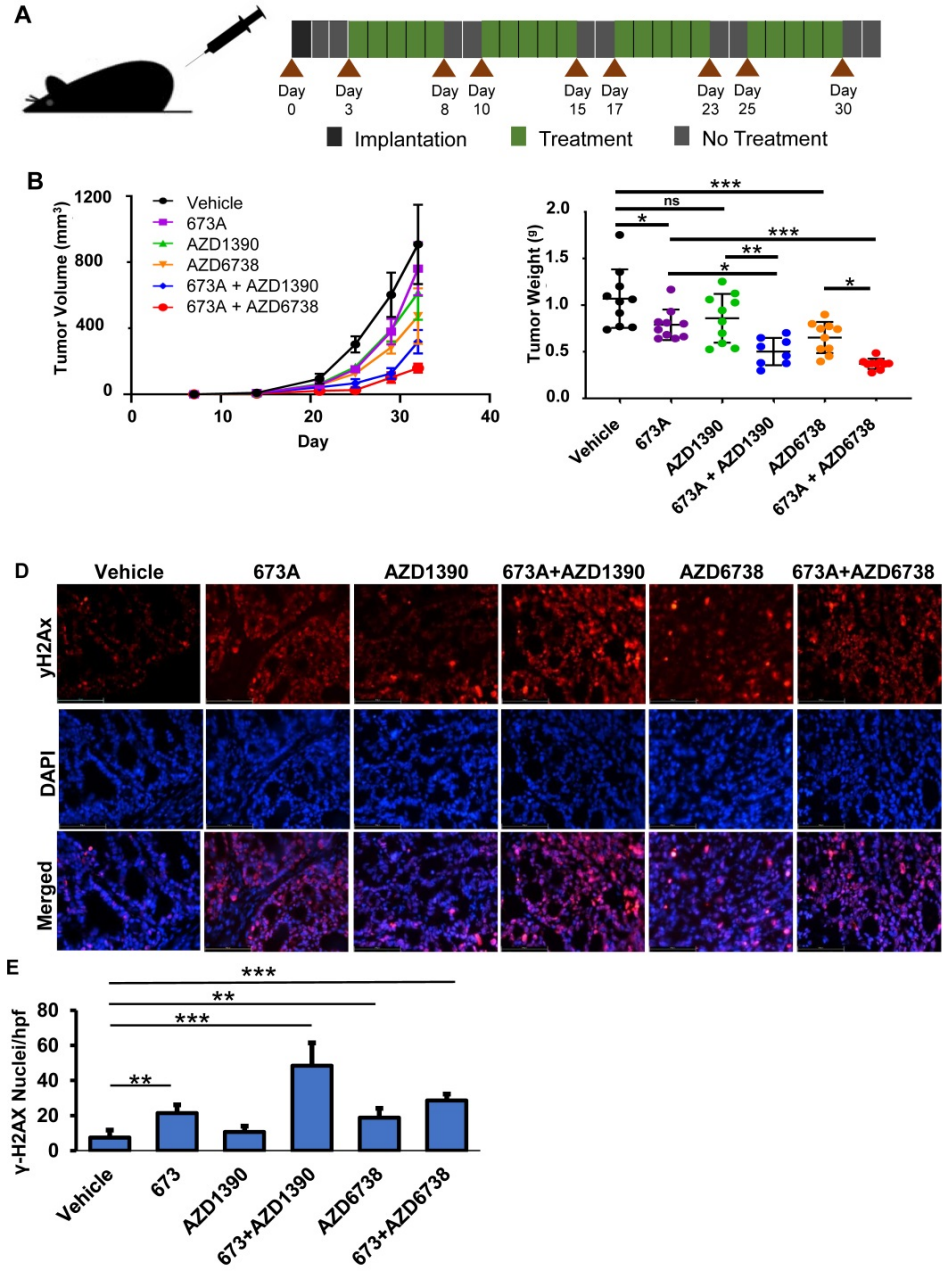
** 673A enhances the effects of ATM/ATR inhibitors in a mouse xenograft model. A)** Diagram of dosing schedule. **B)** Tumor growth curves for mice treated with vehicle, 20 mg/kg 673A, 20 mg/kg AZD1390, 50 mg/kg AZD6738, 20 mg/kg 673A + 20 mg/kg AZD1390, and 20 mg/kg 673A + 50 mg/kg AZD6738. **C)** Plot of final tumor mass. **D)** Immunofluorescence images of γ-H2AX levels in mouse tumors treated with ATM (AZD1390) or ATR inhibitor (AZD6738) alone or in combination with 673A. **E)** Quantification of γ-H2AX positive cells relative to DAPI control. * P ≤ 0.05, ** P ≤ 0.01, *** P ≤ 0.001.

To confirm our *in vitro* finding *in vivo*, OVCAR5 cell were subcutaneously implanted bilaterally in the axilla of NSG mice. The cells were allowed 3 days to engraft before the mice were treated with 673A and/or the ATM or ATR inhibitors. The treatments were given 5 consecutive days per week for 4 weeks (**Figure 5A**). As expected, mice given single agent treatments showed mild to moderate reductions in tumor volume. However, mice treated with both 673A and an ATM or ATR inhibitor had even greater reductions in tumor volume (**Figure 5B**). When control mice reached euthanasia criteria, all animals were euthanized, and tumors were resected and weighted. Confirming tumor volumes, the mice receiving 673A and the ATR inhibitor AZD6738 demonstrated the greatest reduction in tumor weights (**Figure 5C**).

Finally, we evaluated the impact of the ALDHi and/or ATM or ATR inhibitor therapy on DNA damage *in vivo* using γ-H2AX immunofluorescence of the OVCAR5 mouse xenograft tumors. γ-H2AX staining was lowest in the vehicle control (5% γ-H2AX^+^ nuclei), and statistically significantly increased following treatment with 673A (22% γ-H2AX^+^ nuclei) or AZD6738 (18% γ-H2AX^+^ nuclei) (**Figure 5D/E**). Combination therapy of 673A and ATM/ATR inhibitors had higher percentages of γ-H2AX positive nuclei than single agent treatments; 673A+AZD6738 had 30% γ-H2AX positive nuclei, while 673A+AZD1390 had the highest level with 50% γ-H2AX positive nuclei. This data demonstrates the combined inhibition of ALDH and DNA repair checkpoint proteins results in a significant increase in DNA damage, likely due to the being unable to repair DNA damage from the inhibition of ALDH and the subsequent accumulation of DNA damaging aldehydes.

## Discussion

There is growing interest in ALDHi for the treatment of cancer. Indeed a recent phase-II trial of the ALDHi disulfiram combined with chemotherapy in lung cancer patients demonstrated an overall survival advantage 29. As such, the mechanisms whereby ALDHi contribute to cancer cell death are becoming increasingly important to define to facilitate their development and determine which therapies to best combine with ALDHi. Our data defines a novel mechanism by which the ALDH1A family inhibitor, 673A, induces cellular toxicity in ovarian cancer cell lines via the build-up of DNA damaging aldehydes.

The build-up of aldehydes in ovarian cancer cell lines following treatment with 673A is consistent with a study by Pérez-Alea et al, 2017, which demonstrated that treatment of cancer cells with the irreversible isoform-specific ALDH1 inhibitor DIMATE, or down regulation of ALDH1A1 and ALDH1A3, resulted in an accumulation of aldehydes 30. They also demonstrated that ALDH1 inhibition resulted in an increase in cell death and inhibition in tumor growth, however, did not further explore the mechanism of action. This study is consistent with a role of ALDH in protecting hematopoietic stem cells from genotoxic stress 31.

Our study builds on these studies, directly demonstrating ALDH inhibition via 673A, leads to aldehyde accumulation resulting in DNA damage and a proportionate decrease in cell viability. ALDH1A3 knockout studies further confirm a critical role of ALDH1A enzymes in regulating genotoxic aldehydes. However, one CRISPR clone 18-2, despite a 10-fold decrease in ALDH1A3 demonstrated resistance to retinaldehyde induced death. This suggests other factors such as the induction of other dehydrogenases may influence aldehyde sensitivity. Although our data suggests that aldehyde-induced DNA damage is a major mechanism by which ALDH inhibition induces cytotoxicity, we note that aldehyde scavengers were unable to completely rescue 673A-induced DNA damage and cell death, suggesting that other mechanisms of action may work in parallel with aldehyde toxicity. Recently, a study using the novel small molecule inhibitor for ALDH1A, CM37, demonstrated that ALDH1 inhibition in ovarian cancer cell lines caused a significant increase in reactive oxygen species (ROS) 18. ROS induction was correlated with DNA damage, an increase in γ-H2AX, induction of DNA repair genes, and a significant decrease in cell viability. Co-treatment of cells with CM37 and the ROS scavenger Trolox was able to rescue this effect. However, it also is interesting to note that Trolox treatment was only partially protective of CM37- induced ROS, which suggests other factors such as aldehyde accumulation may contribute to DNA damage and reduced cell viability. Consequently, a dual mechanism of ROS and aldehyde-induced DNA damage likely occurs following the inhibition of ALDH enzymes. In addition to this, downstream changes due to lack of RA production will likely contribute 20.

Our study strongly suggests that ALDHi increase cancer cell death in part through the generation of DNA double strand breaks (DSB); ALDHi significantly increase the tail moments of neutral Comet assays which directly measure DSB, ALDHi increase phosphorylation of CHK1 and CHK2 which serve as a DSB checkpoint, and ALDHi synergize with inhibitors ATM and ATR which recognize both direct and replication induced DSBs. Inhibition of ALDH enzymes will lead to an increase in intracellular aldehydes, and aldehyde accumulation is well documented to result in DNA double strand breaks 26. These breaks result in the activation of a wide range of repair enzymes, which determine whether the cell dies or begins the process of DSB repair, via non-homologous end joining (NHEJ) or homologous recombination (HR). Following DNA DSBs, the majority of cells undergo HR, in which ATM and ATR phosphorylate histone H2AX (γ-H2AX) to recruit the repair machinery to the DSB site, and inhibit continuation of the cell cycle via activation of the check point proteins Chk1 and Chk2 32. Eventually, the DNA repair signalling cascade results in the recruitment of BRCA2 and RAD51, which function to repair the DNA DSB. Inhibition of ALDH1A via 673A treatment, likely results in aldehyde-induced DNA DSBs, while combination therapies with ATR and ATM inhibitors prevent the repair of these DSBs, thereby causing cell death. Consequently, this double challenge of inducing DNA DSBs while simultaneously inhibiting DSB repair enzymes results in a synergist decrease on ovarian cancer cell viability. Indeed, our work is consistent with recent studies which showed that PARP inhibitor resistant cells upregulate ALDH1A1 and that co-treatment of ovarian cancer cells with the PARP inhibitor olaparib and an ALDH1A1 inhibitor, resulted in synergistic killing of ovarian cancer cells carrying BRCA2 mutation 33. The synergy between ALDHi and ATM/ATR inhibitors may offer a new therapeutic route for BRCA wild type patients for whom PARP inhibitors are less effective.

In conclusion, we demonstrated a novel mechanism by which ALDH inhibition with 673A resulted in aldehyde-mediated DNA DSB and cellular death of ovarian cancer cell lines. Furthermore, combination therapy with 673A and the DSB repair pathway inhibitors, AZD1390 (ATM) and AZD6738 (ATR), resulted in a synergistic killing ovarian cancer cell lines. One advantage of targeting ATM or ATR in combination with ALDH inhibition is that HGSOC which do not possess HR deficiency, which accounts for 55% of cases 34, should still be responsive to this treatment strategy, while the effectiveness of PARP inhibitors are restricted to cases where HR is known to be impaired. Together, this data suggests ALDHi in combination with the targeting of HR can function as an effective strategy for the treatment of ovarian cancer.

## Supplementary Material

Supplementary figures and tables.

## Figures and Tables

**Table 1:** Combination index analysis for 673A, aldehydes and aldehyde scavengers.

		12.5 μM Retinaldehyde	25 μM 4-HNE	10 μM Hydralazine	100 μM Metformin
**673A**	**0.5 μM**	0.21	0.65	1.9	1.4
	**1 μM**	0.31	0.77	2.3	1.6

Combination indices were calculated, using the Chou-Talalay method, for the indicated doses of 673A, retinaldehyde, 4-HNE, metformin or hydralazine in OVCAR5 cells. CI<1.0 is synergistic, CI>1.0 is antagonistic.

**Table 2:** Combination index analysis for 673A, ATM and ATR inhibitors.

		10 μM AZD1390	1 μM AZD6738
**Conc. 673A (µM)**	**0.625 μM**	0.93	0.25
	**1.25 μM**	0.62	0.28
	**2.5 μM**	0.58	0.4
	**5 μM**	0.37	0.42
	**10 μM**	0.43	0.64
	**20 μM**	0.56	1.2

Combination indices for the indicated doses of 673A and 10 µM AZD1390 or 1 µM AZD6738 in OVCAR5 cells.
