# Supplementary material for: Aldehyde dehydrogenase inhibitors promote DNA damage in ovarian cancer and synergize with ATM/ATR inhibitors
Source: Theranostics. 2021 Jan 20;11(8):3540–51. doi: 10.7150/thno.51885 (PMC7914353; doi:10.7150/thno.51885)
Supplement: Supplementary file 1 — Supplementary figures and tables. [file thnov11p3540s1.pdf]

	FORWARD 5’-3’	REVERSE
**ALDH1A1**	TGTTAGCTGATGGCCGACTTG	TTCTTAGCCCGCTCAACACT
**ALDH1A2**	CCATTGGAGTGTGTGGACAG	GATGAGGGCTCCCATGTAGA
**ALDH1A3**	TCTCGACAAAGCCCTGAAGT	GTCCGATGTTTGAGGAAGGA
**YWHAZ**	ACTTTTGGTACATTGTGGCTTCAA	CCGCCAGGACAAACCAGTAT

Table S1. qPCR primers.

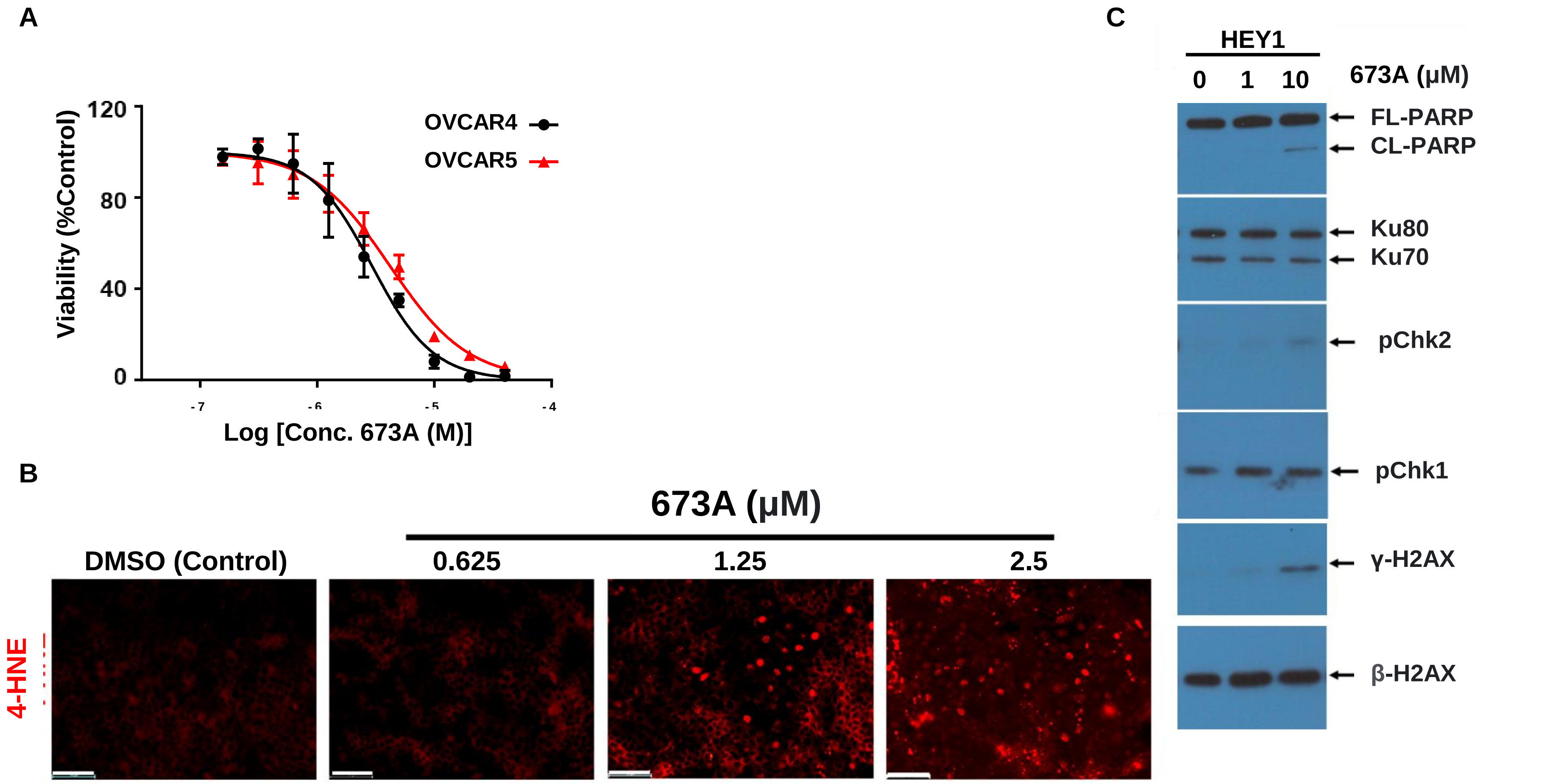

Figure S1. 673A increases 4HNE accumulation and DNA damage. A) OVCAR4 and OVCAR5 cell viability curves. B) IF showing a dose dependent increase in nuclear 4-HNE following treatment with 0.625, 1.25 and 2.5 uM 673A. C) Western Blot of the indicated proteins in HEY1 cells treated with 0, 1 μM or 10 μM 673A.
